# Woodlice of Belgium: an annotated checklist and bibliography (Isopoda, Oniscidea)

**DOI:** 10.3897/zookeys.801.21894

**Published:** 2018-12-03

**Authors:** Pallieter De Smedt, Pepijn Boeraeve, Gert Arijs, Stijn Segers

**Affiliations:** 1 Forest & Nature Lab, Ghent University, Geraardsbergsesteenweg 267, 9090 Melle (Gontrode), Belgium Ghent University Ghent Belgium; 2 SPINICORNIS, Mispeldonk 2, 2820 Bonheiden, Belgium SPINICORNIS Ghent Belgium

**Keywords:** Belgium, macro-detritivores, species distribution, terrestrial isopods

## Abstract

Woodlice are key organisms for nutrient cycling in many terrestrial ecosystems; however, knowledge on this invertebrate group is limited as for other soil fauna taxa. Here, we present an annotated checklist of the woodlice of Belgium, a small but densely populated country in Western Europe. We reviewed all 142 publications on Belgian woodlice, the oldest dating back to 1831 and re-identified all doubtful specimens from the Royal Belgian Institute of Natural Sciences (RBINS) collection. These data is complemented with observations from extensive field surveys dating from March 2014 until December 2017. We report 36 species of woodlice with free-living populations for Belgium. Nine species can be added compared to the latest checklist published in 2000 being *Hyloniscusriparius* (C. Koch, 1838), *Miktoniscuspatiencei* Vandel, 1946, *Trichoniscoidessarsi* Patience, 1908, *Haplophthalmusmontivagus* Verhoeff, 1941, *Porcelliomonticola* Lereboullet, 1853, *Metatrichoniscoidesleydigii* (Weber, 1880), *Trichoniscusalemannicus* Verhoeff, 1917, *Elumacaelata* (Miers, 1877) and *Philosciaaffinis* Verhoeff, 1908. Two species are deleted from the checklist (*Ligidiumgermanicum* Verhoeff, 1901 and *Armadillidiumdepressum* Brandt, 1833) because records are doubtful and no material has been preserved. Additionally the data of the field surveys is used to determine a species status of occurrence in Belgium. For each species, a short overview of their first records is provided and their confirmation as part of the Belgian fauna, their current status, as well as a complete bibliography of the species in Belgium.

## Introduction

Woodlice (Isopoda: Oniscidea) are amongst the largest representatives of the soil invertebrate community in European terrestrial ecosystems (Jeffery et al. 2010). They fragment dead organic material on the forest floor (Anderson 1988; Grelle et al. 2000) and their activity significantly contributes to nutrient cycling in many terrestrial ecosystems (see e.g. David 2014). However, despite their functional importance, they are still poorly studied (David and Handa 2010). In Belgium, distribution data on many species are very scarce (Wouters et al. 2000) in contrast to extensive work in neighbouring countries like the Netherlands (Berg et al. 2008), Great Britain (Gregory 2009), Germany (Grüner 1965) and France (Vandel 1960, 1962, Sechet and Noël 2015). The latest Belgian checklist only reported 27 species with confirmed free-living populations (Wouters et al. 2000) and by comparing this with neighbouring countries it can be assumed that many species could be added to this list (see. e.g. Lock and Durwael 2000, De Smedt et al. 2015, Boeraeve et al. 2017).

A complete overview of the history of woodlice research in Belgium is provided, by checking all existing literature on Belgian woodlice and re-identifying all doubtful or difficult to recognise species present in the collections from the Royal Belgian Institute of Natural Sciences (RBINS). These data are complemented with extensive field surveys carried out from March 2014 until December 2017 in order to produce a new checklist of Belgian woodlice. Additionally the data of the field surveys is used to determine a status of occurrence in Belgium for all species.

**Figure 1.**
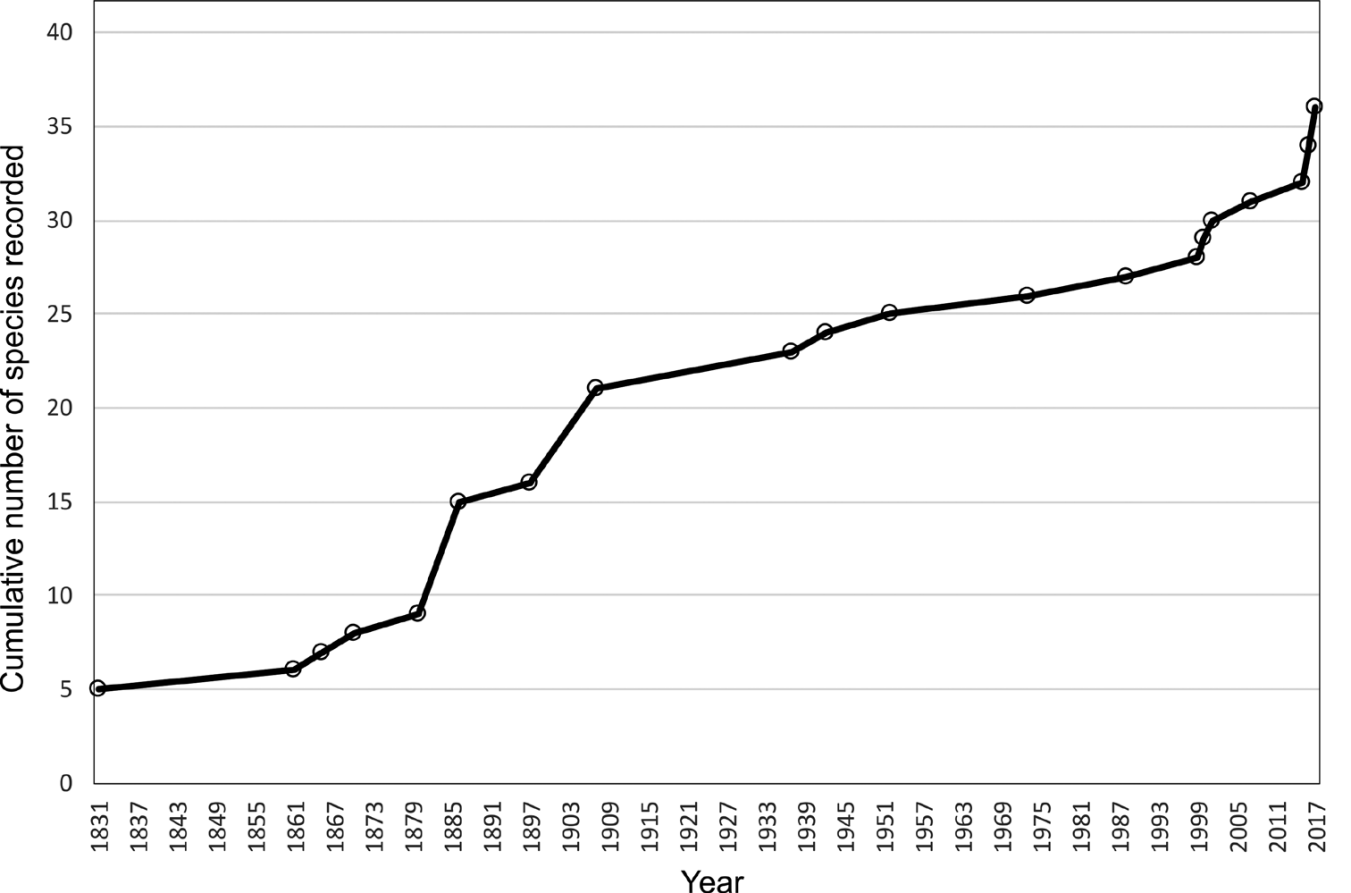
Cumulative number of species published as part of the native fauna of Belgium between 1831 (five species) and 2017 (36 species). Exotic species exclusively living in greenhouses were omitted.

The oldest record of woodlice species in Belgium dates back to 1831 (Carlier 1831) (Fig. 1) and was published in a geographical monograph including all animal species from the province of Liège. This book mentions six species of which one was a synonym of *Armadillidiumvulgare*, which was also mentioned in the list. Carlier (1831) mentions besides the latter species also the three common species being *Oniscusasellus*, *Philosciamuscorum*, and *Porcellioscaber*. The fifth species is *Porcelliolaevis*, which is surprising since the species is extremely rare nowadays in Belgium as in the UK (Harding 2016). Apparently, this species was much more common in previous centuries (see Harding 2016).

During the second half of the 19^th^ century, there was a slow increase in the number of publications and recorded species with nine species in 1870 and the first checklist for Belgium (Plateau 1870) (Fig. 1). From the mid 1880’s until 1910 there was a first peak in woodlouse interest and publications, mostly because of work published by A. Preudhomme de Borre (1886b) and R.S. Bagnall (1907). Preudhomme de Borre (1886b) published a second checklist with 15 species (Fig. 1). In 1910, 21 species were recorded (Fig. 1).

From the 1910’s to the 1970’s, most woodlouse research in Belgium focused on caves (see e.g. all publications by Leruth in the 1930’s and Kersmaekers in the 1970’s). Capart (1942) produced a third checklist but excluded *Ligiaoceanica*, since this species was seen as a marine species by some authors (see e.g., the comments by Pelseneer in 1886). At the time of Capart (1942), 24 species were recorded from Belgium (Fig. 1). In the mid 1950’s and 1960’s, Ph. Polk (see e.g. Polk and Van Oye (1956)) undertook extensive field surveys and identifications and published the first distribution maps for eleven native species (Polk 1957). Despite the new observations, the maps were far from complete and only for a limited number of species. He published a fourth checklist (Polk 1959b) in 1959 but did not really add confirmed species to the list since he claimed *Haplophthalmusdanicus* and *Metatrichoniscoidesleydigii* as new species. Capart (1942) did not mention the first, as he did not cite the publication by Bagnall (1907) when the species was recorded for the first time in Belgium. *M.leydigii* could only be confirmed in 2015 (De Smedt et al. 2016a). Nevertheless, Ph. Polk made an important contribution to the knowledge of Belgian woodlice and compiled the first more extensive bibliography with 33 papers (Polk 1959b). Towards the end of the 20^th^ century a lot of work was summarized under impulse of J.M. Tavernier and K. Wouters who published a fifth checklist, together with a bibliography (Tavernier and Wouters 1989), reporting 27 species that could be validated (Fig. 1). They produced a sixth (Tavernier and Wouters 1991) and seventh (Wouters et al. 2000) checklist but they did not add confirmed species. Wouters et al. (2000) produced an extensive bibliography of 81 papers. After Polk (1957), they were also the second authors to publish distribution maps and this for the 27 native species. However, the distribution data were mostly collected from literature, especially from the extensive but geographically limited field survey by Boon et al. (1993), and from the RBINS collection. Therefore, only few new observations were added, resulting in insufficient data to assess the status of occurrence of woodlice species in Belgium.

At the start of the 21^st^ century, there was a renewed interest in woodlouse research with the discovery of four new species for Belgium by K. Lock (Fig. 1). In 2014, a national terrestrial isopod group “Spinicornis” was founded by the authors of this paper. They aimed to survey the entire territory of Belgium at 10 km × 10 km square resolution by 2020. This led to the discovery of four new species for the fauna of Belgium and finally the confirmation of *M.leydigii* (De Smedt et al. 2016a). This brings the current number of woodlouse species for Belgium at 36 (Fig. 1). Additionally the field surveys undertaken by Spinicornis since 2014 resulted in many new records for almost all Belgian woodlice species. This enables to assess the status and countrywide distribution of all Belgian species for the first time.

## Materials and methods

### Study area

Belgium is a rather small country (ca. 30.500 km²) in Western Europe, but despite its small size, the country shows a rich geology (Pirson et al. 2008). There is a small shoreline (approx. 65 km) and its territory penetrates up to 290 km inland. Along this gradient, the country changes from a largely flat Atlantic region in the north with Holocene and Pleistocene deposits, towards a more continental hilly landscape (up to 694 m elevation) in the east and south with much older (Mesozoic and Palaeozoic) deposits. This varied gradient across such a small country gives the opportunity for many species to establish. This is also the case for woodlice, finding coastal species (Kersmaekers 1988, Lock and Durwael 2000) up to alpine ones (De Smedt et al. 2016b) within this small country.

### Checklist

***Literature***

**Figure 2.**
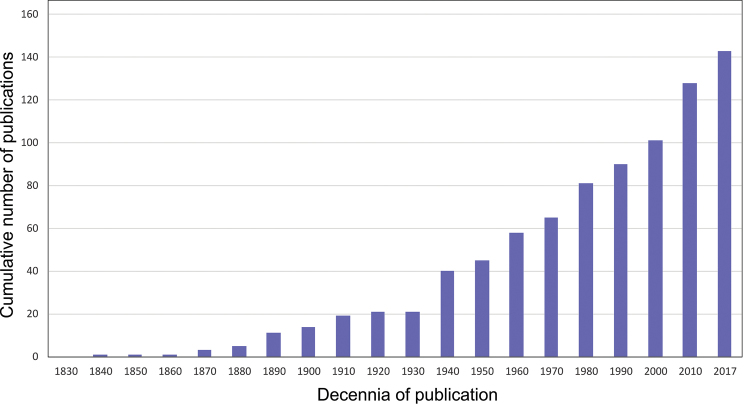
Cumulative number of publications on Belgian woodlice from 1831 until 2017.

All existing literature published or accepted about Belgian woodlice was reviewed if containing distribution data, descriptions, and ecology up to the end of 2017. Our search was based on old bibliographies from Belgium (Polk 1959b, Wouters et al. 2000), all volumes from journals produced by the Royal Belgian Entomological Society (SRBE/KBVE) and through Web of Science and Google Scholar using the keywords [“Belgium” AND “Woodlice”/”Isopod(a)”]. The same searches were carried out for Dutch and French translations [“België” AND “Pissebed(den)”/”Isopod(a)”] and [“Belgique” AND “Cloporte(s)”/“Isopod(a)”]. Subsequently, all articles were scanned on the citing of Belgian woodlice species. Relevant MSc-theses were also included. The retrieved papers are the base for the checklist used to confirm species records by checking original descriptions. All used manuscripts are listed in the bibliography at the end of this paper. Our search resulted in 142 publications of any scientific significance published on Belgian woodlice from 1831 until 2017 (Fig. 2). There has been a steady increase in number of publications since 1830, with only a small dip around World War I. While the first literature records of Belgian woodlice were done in 1831, it increased to 14 publications by the beginning of the 20^th^ century and to 101 at the beginning of the 21^st^ century. Currently, there are 142 publications dealing with Belgian woodlice (Fig. 2).

***Museum collections***

All individuals of 18 species present in the collections of the RBINS were re-identified. *Armadillidiumnasatum*, *A.opacum*, *A.pictum*, *A.pulchellum*, *Cylisticusconvexus*, *Haplophthalmusdanicus*, *H.mengii*, *Philosciamuscorum*, *Porcelliumconspersum*, *Trachelipusrathkii*, *Trichoniscuspusillus*, *T.pygmaeus*, and *Trichoniscoideshelveticus* were checked because these species can easily be misidentified or because closely resembling species were only discovered many years later. *Androniscusdentiger*, *Porcelliodilatatus*, *P.laevis*, *Porcellionidespruinosus*, and *Trichoniscoidesalbidus* were checked because only very limited knowledge is available for these species on both the historical and current distribution and ecology. Records labelled with *Armadillidiumalbum* and *Trichoniscusprovisorius* were not present in the collections. Records of *Ligiaoceanica*, *Ligidiumhypnorum*, *Oniscusasellus*, *Platyarthrushoffmannseggii*, *Armadillidiumvulgare*, *Porcellioscaber*, and *P.spinicornis* are widespread and easy to recognise therefore they are expected to be correct. This re-identification enabled us to check the presence of all species and to verify literature references.

***Field survey and status***

Field surveys were carried out over a four-year period from March 2014 until December 2017 by the authors together with other volunteers from “Spinicornis”. During these surveys, firstly searches for all known Belgian species on locations of old records were carried out, a well as for species that could be expected to occur in Belgium based on their preferred habitat in neighbouring countries.

Secondly, systematic searches of squares of the UTM 10 × 10km grid were carried out in order to map species distribution patterns across Belgium. Main woodlice biotopes were visited in every square. The biotopes visited were (1) an (ancient) forest, (2) a wetland/rivers edge or swamp forest and (3) synantropic habitat (e.g., public park, garden, graveyard…) if present in the 10 km square. These three biotopes cover the habitat niches of most woodlouse species. Additionally, 10 km squares containing a shoreline were surveyed for coastal habitats (e.g., dunes) as well. In some regions, old farms or old quarries have also been visited when present. Woodlice were hand collected by turning stones/dead wood and by sieving the litter layer.

**Figure 3.**
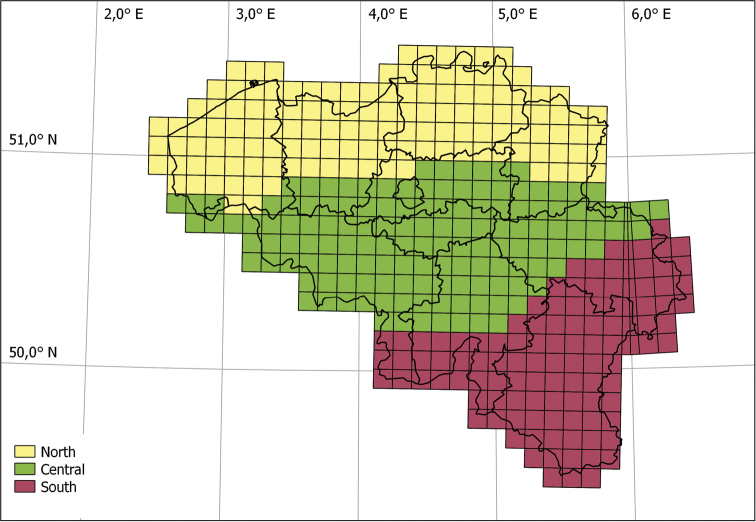
Map of Belgium with the UTM 10×10 km grid. The different colours indicate the different topographical regions used to determine the species status.

By the end of 2017, the field survey campaign has not been completed but enough data has already been collected to assess the current status of occurrence for all species. In order to give a first indication of the distribution pattern this status is not only determined for the complete territory but also for three different topographical regions. The three regions are roughly based on the Belgian topography with lowlands in the north, hilly landscape in the centre and uplands (up to 694 m) in the south (Fig. 3).

Data of the field surveys were used to assess the status of all species but only observations made in sufficiently surveyed squares were withhold. The criterion for a square to be sufficiently surveyed was at least five species recorded in the square. In some parts of Belgium this is about the maximum number of species that can be found so a higher lower-limit would exclude well-searched squares in those parts of Belgium. Records from heated greenhouses and of specimens only identified to genus-level were removed from the dataset. The resulting dataset contains 5110 records from March 2014 until December 2017.

For every region, at least 59.6% of the squares have been sufficiently surveyed, with a total of 254 visited squares out of 381 (66.7%) (Table 1). The field surveys took place across the seasons in every region (Table 2).

The status was assessed based on the number of squares of the UTM 10×10 km grid in which the species was recorded compared to the number of squares that have been surveyed. Six different categories are distinguished from “not present” (0% of the squares) to “very common” (more than 31.5% of the squares) (Table 3).

**Table 1.** Number of squares of the UTM 10×10 km grid per region and number and percentage of squares surveyed between March 2014 and December 2017.

**Region**	**squares in region**	**squares surveyed**	
		#	%
**North**	127	89	70.1
**Centre**	140	97	69.3
**South**	114	68	59.6
**Total**	381	254	66.7

**Table 2.** Number of records per region and per three-month period, corresponding with the seasons of the year.

	**North**	**Centre**	**South**	**Total**
December – February (Winter)	525	617	174	1316
March – May (Spring)	472	395	164	1031
June – August (Summer)	237	469	428	1034
September – November (Autumn)	727	624	378	1729
**Total**	1961	2005	1144	5110

## Results

### Checklist

Since 1831, seven checklists have been published about Belgian woodlice (Plateau 1870, Preudhomme de Borre 1886b, Capart 1942, Polk 1959b, Tavernier and Wouters 1989, 1991, Wouters et al. 2000). The last checklist includes 27 confirmed native species. This checklist adds nine species being *Hyloniscusriparius* (C. Koch, 1838), *Miktoniscuspatiencei* Vandel, 1946, *Trichoniscoidessarsi* Patience, 1908, *Haplophthalmusmontivagus* Verhoeff, 1941, *Porcelliomonticola* Lereboullet, 1853, *Metatrichoniscoidesleydigii* (Weber, 1880), *Trichoniscusalemannicus* Verhoeff, 1917, *Elumacaelata* (Miers, 1877), and *Philosciaaffinis* Verhoeff, 1908. The new checklist below reports 36 species from 19 genera and nine families. Eexotic species that were exclusively found in greenhouses are mentioned with an asterisk (*) but are not counted as Belgian species. New species in bold.

Family Ligiidae

1. *Ligiaoceanica* (Linnaeus, 1767)

2. *Ligidiumhypnorum* (Cuvier, 1792)

Family Trichoniscidae

3. *Androniscusdentiger* Verhoeff, 1908

4. *Haplophthalmusdanicus* Budde-Lund, 1880

5. *Haplophthalmusmengii* (Zaddach, 1844)

6. ***Haplophthalmusmontivagus* Verhoeff, 1941**

7. ***Hyloniscusriparius* (C. Koch, 1838)**

8. ***Metatrichoniscoidesleydigii* (Weber, 1880)**

9. ***Miktoniscuspatiencei* Vandel, 1946**

10. *Trichoniscoidesalbidus* (Budde-Lund, 1880)

11. *Trichoniscoideshelveticus* (Carl, 1908)

12. ***Trichoniscoidessarsi* Patience, 1908**

13. ***Trichoniscusalemannicus* Verhoeff, 1917**

14. *Trichoniscusprovisorius* Racovitza, 1908

15. *Trichoniscuspusillus* Brandt, 1833

16. *Trichoniscuspygmaeus* Sars, 1898

Family Styloniscidae

* *Cordioniscusstebbingi* (Patience, 1907)

Family Oniscidae

17. *Oniscusasellus* Linnaeus, 1758

Family Philosciidae

18. ***Philosciaaffinis* Verhoeff, 1908**

19. *Philosciamuscorum* (Scopoli, 1763)

Family Platyarthridae

20. *Platyarthrushoffmannseggii* Brandt, 1833

* *Trichorhinatomentosa* (Budde-Lund, 1893)

Family Armadillidiidae

21. *Armadillidiumalbum* Dollfus, 1877

22. *Armadillidiumnasatum* Budde-Lund, 1885

23. *Armadillidiumopacum* (C. Koch, 1841)

24. *Armadillidiumpictum* Brandt, 1833

25. *Armadillidiumpulchellum* (Zencker, 1798)

26. *Armadillidiumvulgare* (Latreille, 1804)

27. ***Elumacaelata* (Miers, 1877)**

Family Armadillidae

* *Reductoniscuscostulatus* Kesselyák, 1930

Family Cylisticidae

28. *Cylisticusconvexus* (De Geer, 1778)

Family Porcellionidae

29. *Porcelliodilatatus* Brandt, 1833

30. *Porcelliolaevis* Latreille, 1804

31. ***Porcelliomonticola* Lereboullet, 1853**

32. *Porcellioscaber* Latreille, 1804

33. *Porcelliospinicornis* Say, 1818

34. *Porcellionidespruinosus* (Brandt, 1833)

Family Trachelipodidae

*Naguruscristatus (Dollfus, 1889)

35. *Porcelliumconspersum* (C. Koch, 1841)

36. *Trachelipusrathkii* (Brandt, 1833)

### Field survey and status

**Figure 4.**
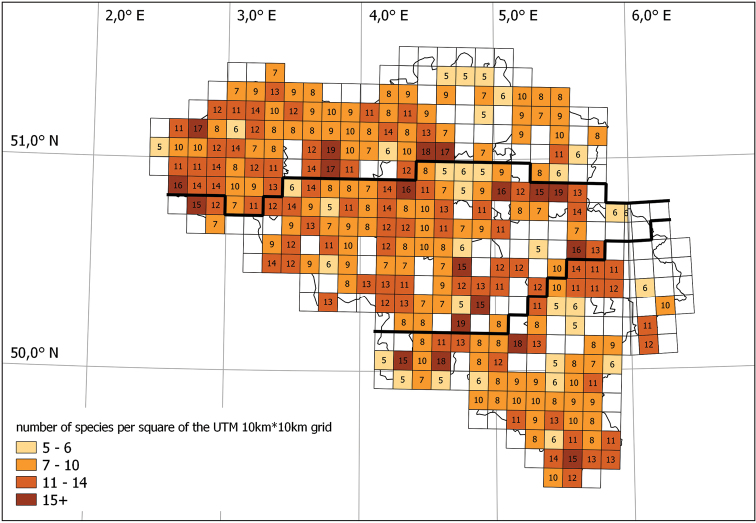
Map of Belgium with the number of species per square of the UTM 10×10 km grid.

During the field surveys, 5110 records of woodlice in Belgium are collected between March 2014 and December 2017. For 35 of the 36 Belgian species there is at least one record in the database (Table 4). Only the species *Miktoniscuspatiencei* was not found during the field surveys. The number of species per square of the UTM 10 × 10 km grid ranged between five and 19 (Fig. 4). At national level, one species was not recorded, six species are very rare, two are rare, three are rather common, fourteen are common and nine are very common (Table 4). Based on percentage occurrence in the number of visited UTM-squares (Table 3) the status of each species per region is given in the discussion.

**Table 3.** Status categories for the Belgian woodlice, together with the lower and upper limits for the percentage of squares where a species was recorded between March 2014 and December 2017 in a certain region.

Status	No. of squares	Rel. no. of squares
Not present	0	0%
Very rare	1–5	< 1.3%
Rare	6–15	1.3–3.9%
Rather common	16–40	3.9–10.5%
Common	41–120	10.5–31.5%
Very common	> 120	> 31.5%

**Table 4.** Number of visited squares where a certain species is recorded and their relative occurrence per region and countrywide.

Species	North		Centre		South		Belgium	
	#	%	#	%	#	%	#	%
* Androniscus dentiger *	9	10.1	44	45.4	18	26.5	71	28.0
* Armadillidium album *	1	1.1	0	0.0	0	0.0	1	0.4
* Armadillidium nasatum *	27	30.3	46	47.4	31	45.6	104	40.9
* Armadillidium opacum *	0	0.0	7	7.2	22	32.4	29	11.4
* Armadillidium pictum *	0	0.0	11	11.3	20	29.4	31	12.2
* Armadillidium pulchellum *	2	2.2	13	13.4	12	17.6	27	10.6
* Armadillidium vulgare *	62	69.7	53	54.6	16	23.5	131	51.6
* Cylisticus convexus *	2	2.2	0	0.0	4	5.9	6	2.4
* Eluma caelata *	3	3.4	0	0.0	0	0.0	3	1.2
* Haplophthalmus danicus *	44	49.4	29	29.9	7	10.3	80	31.5
* Haplophthalmus mengii *	30	33.7	15	15.5	4	5.9	49	19.3
* Haplophthalmus montivagus *	1	1.1	34	35.1	37	54.4	72	28.3
* Hyloniscus riparius *	11	12.4	26	26.8	10	14.7	47	18.5
* Ligia oceanica *	2	2.2	0	0.0	0	0.0	2	0.8
* Ligidium hypnorum *	37	41.6	63	64.9	63	92.6	163	64.2
* Metatrichoniscoides leydigii *	9	10.1	2	2.1	0	0.0	11	4.3
* Miktoniscus patiencei *	0	0.0	0	0.0	0	0.0	0	0.0
* Oniscus asellus *	89	100.0	92	94.8	68	100.0	249	98.0
* Philoscia affinis *	3	3.4	34	35.1	10	14.7	47	18.5
* Philoscia muscorum *	87	97.8	90	92.8	62	91.2	239	94.1
* Platyarthrus hoffmannseggii *	38	42.7	26	26.8	18	26.5	82	32.3
* Porcellio dilatatus *	2	2.2	4	4.1	0	0.0	6	2.4
* Porcellio laevis *	0	0.0	1	1.0	0	0.0	1	0.4
* Porcellio monticola *	0	0.0	0	0.0	3	4.4	3	1.2
* Porcellio spinicornis *	88	98.9	93	95.9	65	95.6	246	96.9
* Porcellio scaber *	65	73.0	77	79.4	62	91.2	204	80.3
* Porcellionides pruinosus *	13	14.6	7	7.2	2	2.9	22	8.7
* Porcellium conspersum *	0	0.0	4	4.1	29	42.6	33	13.0
* Trachelipus rathkii *	37	41.6	29	29.9	8	11.8	74	29.1
* Trichoniscoides albidus *	40	44.9	20	20.6	0	0.0	60	23.6
* Trichoniscoides helveticus *	0	0.0	10	10.3	7	10.3	17	6.7
* Trichoniscoides sarsi *	32	36.0	15	15.5	0	0.0	47	18.5
* Trichoniscus alemannicus *	0	0.0	1	1.0	2	2.9	3	1.2
* Trichoniscus provisorius *	55	61.8	36	37.1	7	10.3	98	38.6
* Trichoniscus pusillus *	69	77.5	64	66.0	55	80.9	188	74.0
* Trichoniscus pygmaeus *	23	25.8	39	40.2	14	20.6	76	29.9

## Discussion

Although many papers have been published on woodlice, many records remained doubtful and the reference collection at the RBINS contained a considerable number of identification errors. Additionally, the number of species recorded in Belgium was relatively low compared to neighbouring countries. This new checklist adds nine species to the last checklist published only 17 years ago (Wouters et al. 2000). In this section, the first record of all species with free-living populations in Belgium is discussed, their current status and a complete bibliography per species is given. The bibliography reports all papers mentioning the particular species. Papers in bold represent the first confirmed Belgian records. Papers in italic include information about the species ecology or distribution.

Certain exotic species are in Belgium only recorded from greenhouses and do not have free-living populations. These species are discussed in a recent paper dedicated to greenhouse species in Belgium (De Smedt et al. 2017a) and only briefly in a separate section of this discussion as they are not considered as part of the Belgian fauna.

### Order Isopoda

#### Suborder Oniscidea

##### Section Diplocheta

###### Family Ligiidae

####### Genus *LIGIA* Fabricius, 1798

######## 1. *Ligiaoceanica* (Linnaeus, 1767)

Van Beneden (1861) first mentioned this species in 1861 as being abundant between stones where they reach the seawater. Since this is a strictly littoral species, certain authors (e.g., Plateau (1870) and Capart (1942)) did not consider it as part of the terrestrial isopod fauna (see e.g., Pelseneer (1886) for a discussion about this). Nevertheless, it is nowadays fully considered as a terrestrial isopod because it can inhabit higher littoral zones and within this genus, there are a few species that are not bound to coastal conditions (Schmalfuss 2003).

**Status**: Coastal species, rare in the north of the country.

**Bibliography: *Van Beneden (1861)***, *Bellynck (1865)*, Pelseneer (1886), *Preudhomme de Borre (1886b)*, *Lameere (1895)*, Maitland (1897), Gilson (1900), Bagnall (1907), *Lameere (1909, 1913, 1931, 1938)*, *Leloup and Miller (1940)*, *Gils (1947)*, *Holthuis (1950)*, *Kesteloot (1956)*, *Lefevere et al. (1956)*, *Leloup and Konietzko (1956)*, *Polk and Van Oye (1956)*, *Leloup (1957)*, *Polk (1959a,b)*, *Leloup et al. (1963)*, *Polk (1963)*, Lefevere (1965), *Polk (1965)*, *Leloup and Polk (1967)*, *Daro (1969)*, *Jocqué and Van Damme (1971)*, *Polk (1976)*, *Van Gompel and Rabaut (1976)*, *Rappé (1977)*, *Eneman (1984)*, *Tavernier and Wouters (1986)*, *Rappé (1989a,b)*, Tavernier and Wouters (1989, 1991), Boon et al. (1993), *Mares (1994)*, Lock and Durwael (2000), *Wouters et al. (2000)*, *Engledow et al. (2001)*, *Jonckheere and Van Rillaer (2001)*, *Huwae and Rappé (2003)*, Maelfait et al. (2004), Vandepitte et al. (2010), Segers (2015), De Smedt et al. (2017b).

####### Genus *LIGIDIUM* Brandt, 1833

######## 2. *Ligidiumhypnorum* (Cuvier, 1792)

Plateau (1870, 1873) was the first to mention the species from Belgium in the 1870’s as *Ligidiumpersoonii* (Brandt) (Plateau 1870) and *Ligidiumagile* (Plateau, 1873). Since this publication, the species has been mentioned in many papers.

**Status**: Very common across the country.

**Bibliography: *Plateau (1870***, *1873)*, Pelseneer (1886), *Preudhomme de Borre (1886b)*, *Lameere (1895)*, Maitland (1897), Bagnall (1907), *Leruth (1937f)*, *Lameere (1938)*, *Leruth (1939)*, Capart (1942), *Polk and Van Oye (1956)*, Polk (1957), *Leloup and Van Meel (1958)*, *Polk (1959a,b)*, *Delhez and Kersmaekers (1973)*, *Kersmaekers and Deroeck (1973)*, *Kersmaekers (1973d)*, *Gysels et al. (1976)*, *Tavernier (1981)*, *Tavernier and Kerwyn (1982)*, Tavernier and Wouters (1989, 1991), *Boon et al. (1993)*, *Branquart et al. (1995)*, *Delhez et al. (1999)*, *Devaere (1999)*, *De Bakker et al. (2000)*, *Schollen (2000)*, *Wouters et al. (2000)*, *Baeté et al. (2003a)*, *Vandekerckhove et al. (2003)*, *Baeté et al. (2004)*, *Dekoninck et al. (2005)*, *Baeté et al. (2006a,b)*, *Van De Vyver (2009)*, *Dethier and Hubart (2010)*, Segers (2015), *De Smedt et al. (2016b,c)*, *Nijs et al. (2016)*, *Boeraeve et al. (2017)*, De Smedt et al. 2018a,*b*).

##### Section Synocheta

###### Superfamily Trichoniscoidea

####### Family Trichoniscidae

######## Genus *ANDRONISCUS* Verhoeff, 1908

######### 3. *Androniscusdentiger* Verhoeff, 1908

Expected to occur in Belgium by Preudhomme de Borre (1886b), but mentioned as *Trichoniscusroseus*. First recorded by Lameere (1897) near Thon-Samson (Namur). Vandel (1933) is the first author to mention the name *A.dentiger*. In the following years, both names are used by different authors. Capart (1942) is the first one to mention both species on his checklist, but indicates that the record of *Trichoniscusroseus* by Lameere (1897) is doubtful and could be *A.dentiger*. Finally, Polk (1957) indicates that the species identified as *T.roseus* is probably *A.dentiger*, and removes *T.roseus* from his checklist. *A.dentiger* specimens from the RBINS were re-identified and all specimens belonged to *A.dentiger* of which the oldest ones dated back to 1916 from Jemelle (Namur) and Schaerbeek (Brussels).

**Status**: Very common in the centre of the country, common in the south and rather common in the north.

**Bibliography**: Moniez (1886), Preudhomme de Borre (1886b), ***Lameere (1897)***, Maitland (1897), *Bagnall (1907, 1908)*, *Vandel (1933)*, *Leruth (1936a,b,c,d,e, 1937b,d,f)*, *Lameere (1938)*, *Leruth (1939)*, Capart (1942), *Polk and Van Oye (1956)*, *Polk* (1957, *1959a,b*), *Delhez and Houssa (1969)*, *Delhez et al. (1973)*, *Delhez and Kersmaekers (1973)*, *Gilson and Hubart (1973)*, *Kersmaekers and Deroeck (1973)*, *Holthuis (1983)*, Tavernier and Wouters (1989, 1991), *Boon et al. (1993)*, *Delhez et al. (1999)*, *Wouters et al. (2000)*, *Dethier and Hubart (2010)*, Segers (2015), *De Smedt et al. (2017a)*.

######## Genus *HAPLOPHTHALMUS* Schöbl, 1860

######### 4. *Haplophthalmusdanicus* Budde-Lund, 1880

First mentioned from greenhouses by Bagnall (1907, 1908), but the species is not incorporated in the checklists from Lameere (1938) and Capart (1942). In 1956, Polk and Van Oye (1956) discovers the species in Ghent and claims the first record, despite citing Bagnall (1907, 1908). The species was discovered in a medieval basement in Brussels (Kersmaekers 1974), but it took until the 21^st^ century for the first confirmed records from wild populations. *H.mengii* samples from the collections of RBINS were re-identified and the oldest samples of *H.danicus* dated back from 2002 (Ramioul, Liège) and 2004 (Cheratte, Liège). However, numerous observations after 2010 proved that the species is much more common than previously thought.

**Status**: Very common in het north and common to rather common in the rest of the country.

**Bibliography: *Bagnall (1907***, *1908)*, *Polk and Van Oye (1956)*, *Polk (1957, 1959a,b)*, *Kersmaekers (1974)*, Tavernier and Wouters (1989,^1991^), Boon et al. (1993), *Wouters et al. (2000)*, *Lock (2007)*, Segers (2015), *De Smedt et al. (2016c, 2017a)*.

######### 5. *Haplophthalmusmengii* (Zaddach, 1844)

First mentioned by Maitland (1897) but unclear if the species was already recorded from Belgium or only from the Netherlands, therefore, the species is mentioned as new for the Belgian fauna by Bagnall (1907). He collected one specimen in a greenhouse in Antwerp. After investigating all museum specimens of *H.mengii*, a specimen collected in 1899 in Han-sur-Lesse (Namur) was discovered. The record consists of one male and one female specimen and is the first record of the species in Belgium. Specimens belonging to *Haplopthalmus mengii/montivagus* were also present in the collections from 1897 and 1898 but it was impossible to identify the species.

**Status**: Very common in het north and common to rather common in the rest of the country.

**Bibliography**: MaiMaitland (1897), ***Bagnall (1907)***, *Leruth (1937a,b,c,e,f, 1939)*, Capart (1942), *Leleup (1948)*, *Polk and Van Oye (1956)*, *Leclercq (1957)*, *Polk (1957, 1959a,b)*, *Delhez et al. (1973)*, *Delhez and Kersmaekers (1973)*, *Kersmaekers and Deroeck (1973)*, Tavernier and Wouters (1989, 1991), *Boon et al. (1993)*, *Branquart et al. (1995)*, *Delhez et al. (1999)*, *Wouters et al. (2000)*, *Lock (2007)*, *Dethier and Hubart (2010)*, Segers (2015), *Nijs et al. (2016)*, De Smedt et al. 2017a.

######### 6. *Haplophthalmusmontivagus* Verhoeff, 1941

First reported record of the species was done by Lock (2007) in 2006. This species closely resembles *H.mengii* and has probably been overlooked for a long time. After checking specimens of *H.mengii* from the collection of the RBINS, *H.montivagus* appeared to be collected in 1998 (Comblain-au-Pont, Liège) and 2002 (Stoumont, Liège).

**Status**: Very common in the centre and south of the country, very rare in the north.

**Bibliography: *Lock (2007)***, Segers (2015), *De Smedt et al. (2016b)*.

######## Genus *HYLONISCUS* Verhoeff, 1908

######### 7. *Hyloniscusriparius* (C. Koch, 1838)

**Figure 5.**
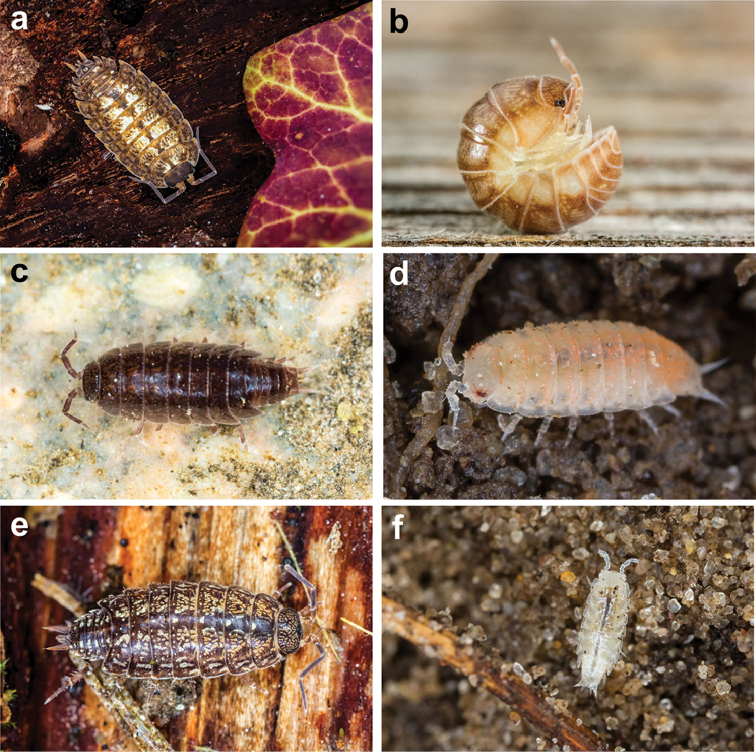
Six of the nine species added to this new checklist, **a***Porcelliomonticola***b***Elumacaelata***c***Hyloniscusriparius***d***Trichoniscoidessarsi***e***Philosciaaffinis* and **f***Metatrichoniscoidesleydigii*; photos: Gert Arijs.

Discovered in Belgium through pitfall trap research in 1998 (Lock and Vanacker 1999). Recent observations indicated that the species is not rare in the country (Fig. 5c). All specimens of *Trichoniscuspusillus* s.l. from the RBINS were re-identified, because *H.riparius* could be easily confused with this species. However, no historical records from *H.riparius* could be discovered.

**Status**: Common across the country.

**Bibliography: *Lock and Vanacker (1999)***, Lock and Durwael (2000), Lock (2001), *Huwae and Rappé (2003)*, Lock (2007), Segers (2015).

######## Genus *METATRICHONISCOIDES* Vandel, 1942

######### 8. *Metatrichoniscoidesleydigii* (Weber, 1880)

Reported by Maitland (1897), but probably this is based on a record from the Netherlands. Polk and Van Oye (1956) found the first individuals of this genus, but the individuals were all females. Identification is only possible by checking male pleopods. Nevertheless, the species was mentioned on all subsequent checklists. A second observation was done in 2009, but it took until 2015 before the first males were observed and the species could be confirmed for the Belgian fauna (De Smedt et al. 2016a) (Fig. 5f).

**Status**: Rather common in the north of the country, very rare in the centre and absent from the south.

**Bibliography**: Maitland (1897), *Polk and Van Oye (1956)*, *Polk (1957, 1959a,b)*, Tavernier and Wouters (1989,^1991^), Boon et al. (1993), *Wouters et al. (2000)*, Segers (2015), ***De Smedt et al. (2016a)***.

######## Genus *MIKTONISCUS* Kesselyák, 1930

######### 9. *Miktoniscuspatiencei* Vandel, 1946

Only two sightings of this species are known in Belgium. After its discovery in 1999 (Lock and Durwael 2000) a second record was done by Lock (2001). In 2015–2016 searches at the same locations where the first two sightings were done could not rediscover the species. The area where the species was found strongly changed through restoration works. It is unclear if the species could be found on other locations in Belgium since the lack of suitable habitat (for details on habitat see Lock and Durwael 2000, Berg et al. 2008).

**Status**: Coastal species, not recorded during the recent field surveys.

**Bibliography: *Lock and Durwael (2000)***, *Lock (2001*, 2007), *Huwae and Rappé (2003)*, Segers (2015), De Smedt et al. (2017b).

######## Genus *TRICHONISCOIDES* Sars, 1898

######### 10. *Trichoniscoidesalbidus* (Budde-Lund, 1880)

Expected to occur in Belgium by Preudhomme de Borre (1886b) and first recorded from Durbuy (Luxembourg) in 1933 by Leruth (1937f). In the collection of the RBINS a male from Rochefort (Namur) in 1929 was discovered, this is probably the first collected individual of this species in Belgium. Records of this species remained extremely scarce until the 21^st^ century.

**Status**: Very common in the north of the country, common in the centre but absent from the south.

**Bibliography**: Preudhomme de Borre (1886b), ***Leruth (1937f***, *1939)*, Capart (1942), *Vandel (1952)*, *Polk and Van Oye (1956)*, *Leclercq (1957)*, *Polk* (1957, *1959a,b*), Tavernier and Wouters (1989, 1991), *Boon et al. (1993)*, *Delhez et al. (1999)*, *Wouters et al. (2000)*, Segers (2015), *De Smedt et al. (2017b*, 2018a,b).

######### 11. *Trichoniscoideshelveticus* (Carl, 1908)

First individuals identified by Vandel (1933) from Jemelle (Namur), but the exact date is unknown. Records of this species before 2010 are very scarce.

**Status**: Absent in the north, rather common in the rest of the country.

**Bibliography: *Vandel (1933***, *1952)*, *Polk and Van Oye (1956)*, *Polk (1957, 1959a,b)*, *Delhez and Kersmaekers (1973)*, *Kersmaekers (1973a)*, *Kersmaekers and Deroeck (1973)*, Tavernier and Wouters (1989, 1991), *Boon et al. (1993)*, *Delhez et al. (1999)*, *Wouters et al. (2000)*, Lock (2001), Segers (2015).

######### 12. *Trichoniscoidessarsi* Patience, 1908

First recorded by Lock (2001) (Fig. 5d). Probably, this species had been overlooked for a long time because of its close resemblance to *T.helveticus*.

**Status**: Very common in the north of the country, common in the centre but absent from the south.

**Bibliography**: Lock and Durwael (2000), ***Lock (2001***, 2007), *Huwae and Rappé (2003)*, Segers (2015), *De Smedt et al. (2017b)*.

######## Genus TRICHONISCUS Brandt, 1833

######### 13. *Trichoniscusalemannicus* Verhoeff, 1917

Discovered in 2015 (De Smedt et al. 2016b), but probably overlooked for a long time because of its close resemblance to *T.pusillus* and *T.provisorius*.

**Status**: Rare in the south of the country, very rare in the centre and absent from the north.

**Bibliography: *De Smedt et al. (2016b)***.

######### 14. *Trichoniscusprovisorius* Racovitza, 1908

First recorded by Kersmaekers (1973c) as a subspecies of *T.pusillus*. Nowadays, no longer considered as a subspecies (Schmalfuss 2003) and can be distinguished from *T.pusillus* by the different shape of the male first pleopod (see e.g. Vandel 1960, De Smedt et al. 2016b). It was not mentioned on the checklists of Tavernier and Wouters (1989, 1991) and only as a subspecies by Wouters et al. (2000). Recordings of this species are extremely scarce in Belgium, since the species was considered a subspecies for a long time. Therefore, all specimens (945 individuals) of *Trichoniscuspusillus* s.l. present at the RBINS were re-identified of which 15 males and 930 females. All males belonged to *T.provisorius*. Interestingly, all male specimens were recorded after 1980. Vandel (1960) reports the species as being expansive and comparing the historical data with the recent surveys it can be assumed that the species is nowadays much more widespread. Historical data from the RBINS collections until 1970 recorded 0% of males across the country while this is 0.04% between 1970 and 2000 and about 1% after 2010.

**Status**: Very common in the north and centre of the country, common in the south.

**Bibliography: *Kersmaekers (1973c)***, *Wouters et al. (2000)*, De Smedt et al. (2015), Segers (2015), *De Smedt et al. (2016b*, De Smedt et al. 2018a,b).

######### 15. *Trichoniscuspusillus* Brandt, 1833

First mentioned by Preudhomme de Borre (1886b), but later on the species appeared to be two species: *T.pusillus* and *T.provisorius*. Except for Kersmaekers (1973c), no author distinguished between the two species. For a sure identification the first male pleopod needs to be examined, but males are extremely rare (about 1.6%) of the population in *T.pusillus* (Vandel 1960). Therefore, identification of this species is often done based on the sex ratio of a large sample of the population (see Fussey 1984, De Smedt et al. 2016b). All *Trichoniscuspusillus* s.l. present in the RBINS collections were re-identified (see *Trichoniscusprovisorius*). No males of *T.pusillus* were detected, but from three localities populations with more than 30 female individuals were recorded and no males were present. These are from Brussels in 1941 (166 ind.), Wanze (Liège) in 1979 (70 ind.) and from Ethe (Luxembourg) in 1981 (109 ind.).

The bibliography presented below should be considered as a bibliography for the species complex *T.alemannicus*/*pusillus*/*provisorius*, except for references from 2015 onwards.

**Status**: Very common across the country.

**Bibliography**: Pelseneer (1886), ***Preudhomme de Borre (1886b)***, *Lameere (1895, 1897)*, Maitland (1897), Bagnall (1907), *Leruth (1937a,b,d,e,f, 1939)*, *Capart (1942)*, *Leleup (1948)*, *Leloup et al. (1954)*, *Polk and Van Oye (1956)*, Polk (1957), *Leloup and Van Meel (1958)*, *Polk (1959a,b)*, *Delhez and Kersmaekers (1973)*, *Kersmaekers and Deroeck (1973)*, *Kersmaekers (1973c)*, *Tavernier (1981)*, *Tavernier and Kerwyn (1982)*, *Holthuis (1983)*, Tavernier and Wouters (1989, 1991), *Boon et al. (1993)*, *Branquart et al. (1995)*, *Delhez et al. (1999)*, Devaere (1999), Lock and Vanacker (1999), Lock and Durwael (2000), *Schollen (2000)*, *Wouters et al. (2000)*, *Lock (2001)*, *Baeté et al.* (2002, *2003a,b, 2004*), *Dekoninck et al. (2005)*, *Baeté et al. (2006a)*, *Loones et al. (2008)*, Dethier and Hubart (2010), De Smedt et al. (2015), Segers (2015), *De Smedt et al. (2016b,c*, 2017a,*b*, De Smedt et al. 2018a,b).

######### 16. *Trichoniscuspygmaeus* Sars, 1898

Bagnall (1907) recorded the first specimens in greenhouses of the Botanical Gardens in Antwerp (Antwerp) and Brussels. A year later, the same author reported free-living populations in Brussels (Bagnall 1908).

**Status**: Very common in the centre of the country, common in the north and the south.

**Bibliography: *Bagnall (1907***, *1908)*, *Vandel (1933)*, *Capart (1942)*, *Polk and Van Oye (1956)*, *Polk* (1957, *1959a,b*, *Kersmaekers (1973c)*, *Kersmaekers and Deroeck (1973)*, Tavernier and Wouters (1989,^1991^), *Boon et al. (1993)*, *Delhez et al. (1999)*, *Wouters et al. (2000)*, Segers (2015), *De Smedt et al. (2016b, 2017a,b)*.

##### Section Crinocheta

###### Superfamily Oniscoidea

####### Family Oniscidae

######## Genus *ONISCUS* Linnaeus, 1758

######### 17. *Oniscusasellus* Linnaeus, 1758

One of the first five species mentioned for the fauna of Belgium by Carlier (1831). From Bellynck (1865) until Preudhomme de Borre (1886b) referred to as *Oniscusmurarius* (Cuvier). No less than 61 publications deal with this species, making it the third most cited species in Belgian literature references.

**Status**: Very common across the country.

**Bibliography: *Carlier (1831)***, *Bellynck (1865)*, *Plateau (1870)*, Pelseneer (1886), *Plateau (1886)*, *Preudhomme de Borre (1886b)*, *Lameere (1895, 1897)*, Maitland (1897), *Schouteden (1901)*, Bagnall (1907), *Leruth (1937f)*, *Lameere (1938)*, *Leruth (1939)*, Capart (1942), *Leleup (1948)*, *Leloup et al. (1954)*, *Polk and Van Oye (1956)*, *Leclercq (1957)*, *Polk (1957, 1959a,b*), *Kersmaekers and Deroeck (1973)*, *Kersmaekers (1973c)*, *Gysels et al. (1976)*, *Tavernier (1981)*, *Tavernier and Kerwyn (1982)*, *Holthuis (1983)*, Tavernier and Wouters (1989, 1991), *Boon et al. (1993)*, *Branquart et al. (1995)*, *Lambrechts (1997)*, *Delhez et al. (1999)*, *Devaere (1999*), *Boon and Wijns (2000)*, *De Bakker et al. (2000)*, *Schollen (2000)*, *Wouters et al. (2000)*, *Baeté et al. (2002, 2003a,b)*, *Huwae and Rappé (2003)*, *Hendrickx et al. (2003)*, *Vandekerckhove et al. (2003)*, *Baeté et al. (2004)*, *Dekoninck et al. (2005)*, *Baeté et al. (2006a,b)*, *Loones et al. (2008)*, *Van De Vyver (2009)*, *Dethier and Hubart (2010)*, Segers (2015), *De Smedt et al. (2016b,c)*, *Nijs et al. (2016)*, *Boeraeve et al. (2017)*, *De Smedt et al. (2017a,b, 2018a*,b).

####### Family Philosciidae

######## Genus *PHILOSCIA* Latreille, 1804

######### 18. *Philosciaaffinis* Verhoeff, 1908

Expected to occur in Belgium by De Smedt et al. (2015) and shortly afterwards discovered in 2014 (Boeraeve et al. 2017) (Fig. 5e). Boeraeve et al. (2017) checked all individuals present in the collection of the RBINS and discovered that the species was already collected in Belgium in 1938 but misidentified as *P.muscorum*. In total, they discovered eight historic records. The species proved to be widespread in Belgium and was recorded in eight out of ten provinces after 2014.

**Status**: Very common in the centre of the country, common in the south and rare in the north.

**Bibliography**: De Smedt et al. (2015), Segers (2015), ***Boeraeve et al. (2017)***.

######### 19. *Philosciamuscorum* (Scopoli, 1763)

One of the five first species mentioned for the fauna of Belgium by Carlier (1831) as *Philosciasylvestris* (Latr.). This is the second most cited species in Belgian woodlouse literature with 63 publications mentioning the species.

**Status**: Very common across the country.

**Bibliography: *Carlier (1831)***, Bellynck (1865), *Plateau (1870, 1873)*, Pelseneer (1886), Plateau (1886), *Preudhomme de Borre (1886b)*, *Lameere (1895, 1897)*, Maitland (1897), *Schouteden (1901)*, Bagnall (1907), *Leruth (1937f)*, *Lameere (1938)*, *Leruth (1939)*, Capart (1942), *Leleup (1948)*, *Leloup et al. (1954)*, *Leloup and Konietzko (1956)*, *Polk and Van Oye (1956)*, *Polk* (1957, *1959a,b*), *Dumont and Gysels (1971)*, *Kersmaekers and Deroeck (1973)*, *Kersmaekers (1973c)*, *Gysels et al. (1976)*, *Tavernier (1981)*, *Tavernier and Kerwyn (1982)*, *Holthuis (1983)*, Tavernier and Wouters (1989, 1991), *Boon et al. (1993)*, *Branquart et al. (1995)*, *Lambrechts (1997)*, *Devaere (1999)*, *De Bakker et al. (2000)*, *Lock and Durwael (2000)*, *Schollen (2000)*, *Wouters et al. (2000)*, *Lock (2001)*, *Baeté et al.* (2002, *2003a,b*), *Huwae and Rappé (2003)*, *Hendrickx et al. (2003)*, *Vandekerckhove et al. (2003)*, *Baeté et al. (2004)*, Maelfait et al. (2004), *Dekoninck et al. (2005)*, *Baeté et al. (2006a)*, *Loones et al. (2008)*, *Van De Vyver (2009)*, *Dethier and Hubart (2010)*, Segers (2015), *De Smedt et al. (2016b,c)*, *Nijs et al. (2016)*, *Boeraeve et al. (2017)*, *De Smedt et al. (2017a,b, 2018a*,b).

####### Family Platyarthridae

######## Genus *PLATYARTHRUS* Brandt, 1833

######### 20. *Platyarthrushoffmannseggii* Brandt, 1833

First recorded by Mac Leod (1880), and appeared to be common but undersampled (Lameere 1897, Adam and Leloup 1940) because the unusual habitat (ant nests) for a woodlouse. This is the only myrmecophilous woodlouse species in Belgium.

**Status**: Very common in the north of the country, common in the centre and south.

**Bibliography: *Mac Leod (1880)***, *Moniez (1886)*, Pelseneer (1886), *Preudhomme de Borre (1886b)*, *Lameere (1895, 1897)*, Maitland (1897), *Schouteden (1901)*, Bagnall (1907), *Massart (1912)*, *Collart (1936)*, *Lameere (1938)*, *Adam and Leloup (1940)*, Capart (1942), *Polk and Van Oye (1956)*, *Polk* (1957, *1959a,b)*, *Kersmaekers and Deroeck (1973)*, *Tavernier (1981)*, Tavernier and Wouters (1989, 1991), *Boon et al. (1993)*, *Wouters et al. (2000)*, *Dekoninck et al. (2007)*, Segers (2015), *De Smedt et al. (2017a)*, *Parmentier et al. (2017)*.

###### Superfamily Armadilloidea

####### Family Armadillidiidae

######## Genus *ARMADILLIDIUM* Brandt, 1833

######### 21. *Armadillidiumalbum* Dollfus, 1877

Discovered by Kersmaekers (1988), which is the only published faunistical record so far, but it was also recorded during our field surveys. The species is both mentioned on the marine and brackish water isopod checklist (Rappé 1989a) as on terrestrial isopod checklists (Tavernier and Wouters 1989, 1991, Wouters et al. 2000), because its restriction to coastal habitat.

**Status**: Coastal species, very rare in the north of the country.

**Bibliography: *Kersmaekers (1988)***, *Rappé (1989a)*, Tavernier and Wouters (1989, 1991), Boon et al. (1993), Lock and Durwael (2000), *Wouters et al. (2000)*, *Huwaé and Rappé (2003)*, *Maelfait et al. (2004)*, Hoffmann (2006), Segers (2015), De Smedt et al. (2017b).

######### 22. *Armadillidiumnasatum* Budde-Lund, 1885

Expected to occur in Belgium by Preudhomme de Borre (1886b) and first discovered by Bagnall (1907) in greenhouses in Brussels and Antwerp. In the collections of the RBINS records from 1941 and 1943 from the museum gardens and on a roadside verge are present, both anthropogenic environments. It took until 1972 before the first non-anthropogenic populations were discovered in the southern part of the country (Kersmaekers 1972).

**Status**: Very common in the centre and the south of the country, common in the north.

**Bibliography**: Preudhomme de Borre (1886b), Maitland (1897), ***Bagnall (1907, 1908)***, *Capart (1942)*, *Polk and Van Oye (1956)*, *Polk (1957, 1959a,b)*, *Kersmaekers (1972)*, *Kersmaekers and Deroeck (1973)*, Tavernier and Wouters (1989, 1991), *Boon et al. (1993)*, *Wouters et al. (2000)*, Huwae and Rappé (2003), Segers (2015), *De Smedt et al. (2017a)*.

######### 23. *Armadillidiumopacum* (C. Koch, 1841)

First mentioned by Preudhomme de Borre (1886b) as *A.sulcatum*, but he corrected the identification later on to *A.opacum* (Preudhomme de Borre 1886a, Capart 1942). Nevertheless, *A.sulcatum* instead of *A.opacum* was reported on the checklist of Maitland (1897) and by Bagnall (1907). The oldest individuals that could be re-identified from the RBINS collections were collected by A. Capart in the 1940’s.

**Status**: Very common in the south of the country, rather common in the centre and absent in the north.

**Bibliography**: Pelseneer (1886), ***Preudhomme de Borre*** (*1886a*,**b**), Maitland (1897), Bagnall (1907), Capart (1942), *Polk and Van Oye (1956)*, *Polk (1957, 1959a,b)*, *Gysels et al. (1976)*, Tavernier and Wouters (1989, 1991), *Boon et al. (1993)*, *Devaere (1999)*, *Wouters et al. (2000)*, *Vandekerckhove et al. (2003)*, *Dekoninck et al. (2005)*, Segers (2015), *Nijs et al. (2016)*, *De Smedt et al. (2018a*,b).

######### 24. *Armadillidiumpictum* Brandt, 1833

Mentioned for the first time for Belgium by Plateau (1870) but the species was re-identified by Preudhomme de Borre (1886) as being *A.pulchellum*. Additionally, Bagnall (1907) mentioned the species as occurring in Belgium but without any reference. Leruth (1937f) could therefore be the first one to record the species from Belgium. Belgian specimens from the RBINS were re-identified, which mostly originated from the surveys done by Capart (1942), and found both *A.pictum* and *A.pulchellum* in the samples. Both species are easily confused and historical records without preserved animals should be treated with caution.

**Status**: Common in the centre and south of the country, absent from the north.

**Bibliography**: *Plateau (1870)*, Preudhomme de Borre (1886b), Bagnall (1907), ***Leruth (1937f***, *1939)*, Capart (1942), *Polk and Van Oye (1956)*, *Polk (1957, 1959a,b)*, *Kersmaekers and Deroeck (1973)*, *Gysels et al. (1976)*, *Holthuis (1983)*, Tavernier and Wouters (1989, 1991), Boon et al. (1993), *Wouters et al. (2000)*, *Dekoninck et al. (2005)*, *Dethier and Willems (2005)*, Segers (2015), *De Smedt et al. (2016b)*.

######### 25. *Armadillidiumpulchellum* (Zencker, 1798)

Preudhomme de Borre (1886b) re-identified the specimens collected by Plateau (1870) and concluded that the species under consideration was *A.pulchellum* and not *A.pictum*. This is the first record of the species for Belgium. However, the species is easily confused with *A.pictum* (see section on *A.pictum* for additional information).

**Status**: Common in the centre and south of the country, and rare in the north.

**Bibliography: *Preudhomme de Borre*** (*1886a*,**b**), Pelseneer (1886), *Lameere (1895, 1897)*, Maitland (1897), Bagnall (1907), Capart (1942), *Polk and Van Oye (1956)*, *Polk (1957, 1959a,b)*, *Gysels et al. (1976)*, Tavernier and Wouters (1989, 1991), Boon et al. (1993), *Devaere (1999)*, De Bakker et al. (2000), *Wouters et al. (2000)*, *Vandekerckhove et al. (2003)*, *Dekoninck et al. (2005)*, Segers (2015)

######### 26. *Armadillidiumvulgare* (Latreille, 1804)

One of the five first species on the Belgian list (Carlier 1831), Carlier (1831) mentions two species (*Armadillovulgaris* Latr. and *Armadillovariegatus* Latr.) that eventually proved to be the same species (Schmalfuss 2003). Bellynck (1865) mentions *Armadillotriviale*, which also proves to be a synonym of *A.vulgare* (Schmalfuss 2003). Plateau (1870) reports both *Armadillidiumvulgare* and *Armadillidiumtriviale*. Preudhomme de Borre (1886b) and Maitland (1897) mention *A.triviale* or *A.trivialis* as a subspecies of *A.vulgare*. This was also supported by Capart (1942). Afterwards, only *A.vulgare* has been mentioned in the Belgian literature. Interesting is the record by Troubleyn et al. (2009) from the remains of two woodlice, one unidentified woodlouse and the other one being *A.vulgare*, that were found in cesspits of on old prison at the main square of Malines dating back to the 14^th^ century. This is the oldest record of a woodlouse in Belgium.

**Status**: Very common in the north and the centre of the country, common in the south.

**Bibliography: *Carlier (1831)***, Bellynck (1865), *Plateau (1870)*, Pelseneer (1886), *Plateau (1886)*, *Preudhomme de Borre* (1886a,*b*), *Lameere (1895, 1897)*, Maitland (1897), *Schouteden (1901)*, Bagnall (1907), *Senden (1936)*, *Lameere (1938)*, Capart (1942), *Leleup (1948)*, *Leloup and Konietzko (1956)*, *Polk and Van Oye (1956)*, *Polk* (1957, *1959a,b), Kersmaekers and Deroeck (1973)*, *Gysels et al. (1976)*, *Tavernier (1981)*, Tavernier and Wouters (1989, 1991), *Boon et al. (1993)*, *Branquart et al. (1995)*, *Lambrechts (1997)*, *Lock and Vanacker (1999)*, *Lock and Durwael (2000)*, *Schollen (2000)*, *Wouters et al. (2000)*, *Lock (2001)*, *Baeté et al. (2003a)*, *Huwae and Rappé (2003)*, *Vandekerckhove et al. (2003)*, Maelfait et al. (2004), *Troubleyn et al. (2009)*, *Van De Vyver (2009)*, *Dethier and Hubart (2010)*, Segers (2015), Nijs et al. (2016), *De Smedt et al. (2017a,b, 2018a*,b).

######## Genus *ELUMA* Budde-Lund, 1885

######### 27. *Elumacaelata* (Miers, 1877)

Discovered for the first time in Belgium in 2016 (De Smedt et al. 2017b) (Fig. 5b). The species was expected to occur in Belgium since its discovery in the Netherlands close to the Belgian border (Lock and Durwael 2000), but it took more than 20 years since its first sighting in the Netherlands, to find the first Belgian specimens. It is still unclear if the species is truly native or naturalised in Belgium after colonisation from the Netherlands, where it could be accidentally introduced (De Smedt et al. 2017b). Berg et al. (2008) mentions the species from Belgium based on a reference of Lock in 2000, but this publication does not exist and can be classified as a typo.

**Status**: Rare in the north of the country, absent from the centre and the south.

**Bibliography**: Lock and Durwael (2000), *Huwae and Rappé (2003)*, ***De Smedt et al. (2017b)***, Boeraeve et al. (2017).

####### Family Cylisticidae

######## Genus *CYLISTICUS* Schnitzler, 1853

######### 28. *Cylisticusconvexus* (De Geer, 1778)

Expected to occur in Belgium by Preudhomme de Borre (1886b) and recorded for the first time in the same year by Moniez (1886). Records of this species have always been scarce with a peak during field research from Capart (1942); he collected specimens from at least six locations in the south of the country. Our recent observations indicate that the species is still scarce in the south of the country but was discovered as some isolated populations in the north as well.

**Status**: Rather common in the south of the country, rare in the north and absent from the centre.

**Bibliography**: Preudhomme de Borre (1886b), ***Moniez (1886)***, Bagnall (1907), *Leruth (1937f, 1939)*, Capart (1942), *Polk and Van Oye (1956)*, *Polk* (1957, *1959a,b), Kersmaekers and Deroeck (1973)*, Tavernier and Wouters (1989,^1991^), Boon et al. (1993), *Wouters et al. (2000)*, Segers (2015), De Smedt et al. (2017a).

####### Family Porcellionidae

######## Genus *PORCELLIO* Latreille, 1804

######### 29. *Porcelliodilatatus* Brandt, 1833

First mentioned by Plateau (1870), but according to Preudhomme de Borre (1886b), the identifications by Plateau (1870) were not correct and appeared to be *P.scaber* (see also Plateau 1886). Therefore, Preudhomme de Borre (1886) does the first record in 1886. Re-identification of specimens from the RBINS dated back to 1898 from Charleroi (Hainaut). Sightings of the species are very rare and mostly associated to manmade structures like old horse and cow stables.

**Status**: Rather common in the centre of the country, rare in the north and absent from the south.

**Bibliography**: *Plateau (1870)*, Pelseneer (1886), *Plateau (1886)*, ***Preudhomme de Borre (1886b)***, *Lameere (1895)*, Maitland (1897), Bagnall (1907), *Leruth (1937f, 1939)*, *Capart (1942)*, *Polk and Van Oye (1956)*, *Polk* (1957, *1959a,b), Kersmaekers and Deroeck (1973)*, *Holthuis (1983)*, Tavernier and Wouters (1989, 1991), *Boon et al. (1993)*, *Delhez et al. (1999)*, *Wouters et al. (2000)*, Segers (2015), De Smedt et al. (2017a).

######### 30. *Porcelliolaevis* Latreille, 1804

One of the first five species mentioned for the country by Carlier (1831). He mentions that the species could be found frequently under stones. Records from the 20^th^ century are extremely rare and the only literature records are from Schouteden (1901), Polk and Van Oye (1956) and Boon et al. (1993). In the collection of the RBINS some individuals collected in Belgium in 1916 in Leuven (Flemish-Brabant) and the 1940’s in the Museum Garden (a zoo at that time) (Brussels) were found. Since 2000, the species has only been recorded from Wellen (Limburg) in 2015 in an old horse stable. However, despite an intensive search in 2017, after the buildings at the site were renovated, the species could not be rediscovered.

**Status**: Very rare in the centre, absent from the rest of the country.

**Bibliography: *Carlier (1831)***, *Plateau (1870)*, Pelseneer (1886), *Preudhomme de Borre (1886a,b)*, *Lameere (1895,^1897^)*, Maitland (1897), *Schouteden (1901)*, Bagnall (1907), Capart (1942), *Polk and Van Oye (1956)*, *Polk* (1957, *1959a,b),*Tavernier and Wouters (1989,^1991^), *Boon et al. (1993)*, *Wouters et al. (2000)*, Segers (2015).

######### 31. *Porcelliomonticola* Lereboullet, 1853

Expected to occur in Belgium by Preudhomme de Borre (1886b) (mentioned as *Porcelliolugubris*), but only recently discovered in Belgium in 2014 (De Smedt et al. 2015) (Fig. 5a).

**Status**: Rather common in the south, absent from the rest of the country.

**Bibliography**: Preudhomme de Borre (1886b), ***De Smedt et al. (2015)***, Segers (2015), Boeraeve et al. (2017).

######### 32. *Porcellioscaber* Latreille, 1804

One of the first five species mentioned for the fauna of Belgium by Carlier (1831). This species is mentioned in 64 publications on Belgian woodlice, making it the most cited species.

**Status**: Very common across the country.

**Bibliography: *Carlier (1831)***, Bellynck (1865), *Plateau (1870)*, Pelseneer (1886), *Plateau (1886)*, *Preudhomme de Borre (1886b)*, *Lameere (1895, 1897)*, Maitland (1897), *Schouteden (1901)*, Bagnall (1907), *Senden (1936)*, *Leruth (1937f)*, *Lameere (1938)*, *Leruth (1939)*, Capart (1942), *Leleup (1948)*, *Leloup et al. (1954)*, *Leloup and Konietzko (1956)*, *Polk and Van Oye (1956)*, *Polk* (1957, *1959a,b), Delhez and Kersmaekers (1973)*, *Kersmaekers and Deroeck (1973)*, *Gysels et al. (1976)*, *Tavernier (1981)*, *Holthuis (1983)*, Tavernier and Wouters (1989, 1991), *Donker and Bogert (1991)*, *Donker (1992)*, *Boon et al. (1993)*, *Donker et al. (1993)*, *Branquart et al. (1995)*, *Lambrechts (1997)*, *Delhez et al. (1999)*, *Devaere (1999)*, *Lock and Vanacker (1999)*, *Lock and Durwael (2000)*, *Schollen (2000)*, *Wouters et al. (2000)*, *Lock (2001)*, *Baeté et al. (2003a)*, *Huwae and Rappé (2003)*, *Hendrickx et al. (2003)*, *Vandekerckhove et al. (2003)*, *Baeté et al. (2004)*, Maelfait et al. (2004), *Dekoninck et al. (2005)*, *Dethier and Willems (2005)*, *Baeté et al. (2006a)*, *Swiecicka and Mahillon (2006)*, *Loones et al. (2008)*, *Van De Vyver (2009)*, *Dethier and Hubart (2010)*, De Smedt et al. (2015), Segers (2015), *De Smedt et al. (2016b,c)*, *Nijs et al. (2016)*, *Boeraeve et al. (2017)*, *De Smedt et al. (2017a, b, 2018a*, b).

######### 33. *Porcelliospinicornis* Say, 1818

First mentioned by Bellynck (1865) with the French name “Porcellion peint”. The first checklist by Plateau (1870) refers to the publication of Bellynck (1865) as the only observation up to that date. Afterwards, almost exclusively recorded from anthropogenic habitats.

**Status**: Very common across the country.

**Bibliography: *Bellynck (1865)***, *Plateau (1870)*, Pelseneer (1886), *Preudhomme de Borre (1886b)*, *Lameere (1895)*, Maitland (1897), Bagnall (1907), *Leruth (1937f, 1939)*, Capart (1942), *Polk and Van Oye (1956)*, *Polk* (1957, *1959a,b), Kersmaekers and Deroeck (1973)*, *Gysels et al. (1976)*, *Holthuis (1983)*, Tavernier and Wouters (1989, 1991), Tavernier and Wouters (1991), *Boon et al. (1993)*, *Wouters et al. (2000)*, *Dethier and Willems (2005)*, Segers (2015), De Smedt et al. (2015, 2017a).

######## Genus *PORCELLIONIDES* Miers, 1877

######### 34. *Porcellionidespruinosus* (Brandt, 1833)

First observations from the 1870’s and first mentioned by Preudhomme de Borre (1886b). Observations are scattered and Boon et al. (1993) carried out the bulk of the observations during an intensive field survey. They found the species in most of the old stables and compost heaps they visited. The species is always associated with anthropogenic environments (compost heaps, graveyards, old stables…).

**Status**: Common in the north of the country, rather common in the centre, and rare in the south.

**Bibliography: *Preudhomme de Borre (1886b)***, Pelseneer (1886), *Lameere (1895, 1897)*, Maitland (1897), *Schouteden (1901)*, Bagnall (1907), Capart (1942), *Polk and Van Oye (1956)*, *Polk* (1957, *1959a,b), Holthuis (1983)*, Tavernier and Wouters (1989, 1991), *Boon et al. (1993)*, *Wouters et al. (2000)*, Segers (2015), De Smedt et al. (2017a).

####### Family Trachelipodidae

######## Genus *PORCELLIUM* Dahl, 1916

######### 35. *Porcelliumconspersum* (C. Koch, 1841)

First recorded by Capart (1942) in 1941 and confirmed based on individuals stored in the RBINS collections. Records remain very scarce until 2014, but targeted research shows that the species is more common than observed from the few records.

**Status**: Very common in the south of the country, rather common in the centre and absent from the north.

**Bibliography: *Capart (1942)***, *Polk and Van Oye (1956)*, *Polk (1957, 1959a,b)*, *Kersmaekers (1974)*, Tavernier and Wouters (1989, 1991), Boon et al. (1993), *Branquart et al. (1995)*, *Wouters et al. (2000)*, *Dekoninck et al. (2005)*, Segers (2015).

######## Genus *TRACHELIPUS* Budde-Lund, 1908

######### 36. *Trachelipusrathkii* (Brandt, 1833)

First mentioned by Preudhomme de Borre (1886b) as being common in the country. Since the species can easily be confused with e.g. different *Porcellio* species, all material present at the RBINS collections was re-examined (286 individuals from 78 records). However, no misidentifications could be detected. The oldest individuals were from Leuven (Flemish-Brabant) in 1916.

**Status**: Very common in the north of the country, common in the centre and in the south.

**Bibliography**: Pelseneer (1886), ***Preudhomme de Borre (1886b)***, *Lameere (1895)*, Maitland (1897), Bagnall (1907), Capart (1942), *Leleup (1948)*, *Polk and Van Oye (1956), Polk* (1957, *1959a,b), Kersmaekers and Deroeck (1973)*, *Tavernier (1981)*, *Tavernier and Kerwyn (1982)*, Tavernier and Wouters (1989, 1991), *Boon et al. (1993)*, *Devaere (1999)*, *Lock and Vanacker (1999)*, *Wouters et al. (2000)*, *Huwae and Rappé (2003)*, *Dekoninck et al. (2005)*, *Van De Vyver (2009)*, De Smedt et al. (2015), Segers (2015), *Nijs et al. (2016)*, *De Smedt et al. (2017b, 2018a*,b).

### Deleted species

Six species were mentioned on at least one of the previous checklists, but are not present anymore on the current checklist. Most species appeared to be misidentifications or could not be confirmed because material was not preserved and literature citings are incomplete.

*Androniscusroseus* (C. Koch, 1838) was first mentioned by Lameere (1897), but after much of confusion between this species and *Androniscusdentiger* by different authors mentioning one of the two species, it became clear that only *A.dentiger* was recorded from Belgium (Polk 1957) (see *Androniscusdentiger* above).

*Armadillidiumdepressum* Brandt, 1833 was first mentioned by Tavernier and Wouters (1989). The species was apparently collected on a graveyard in the province of East-Flanders near Ninove. However, the species could not be verified and even if the identification is correct the species can be assumed as imported e.g. from Great Britain where the species is common in the south (Gregory 2009) and the species has no current free-living populations in Belgium. Extensive searches for woodlice on Belgian graveyards did not reveal the presence of the species. The species was included in the checklists from Tavernier and Wouters (Tavernier and Wouters 1989, 1991, Wouters et al. 2000) and Huwae and Rappé (2003) mentioned the species based on the same references. Baeté et al. (2003b) found the species in the nature reserve Walenbos (Flemish-Brabant), but later on, this appeared to be *A.opacum*. Finally, De Smedt et al. (2015) propose to remove the species from the Belgian list.

*Armadillidiumsulcatum* Milne-Edwards, 1840 is a species from northern Algeria (Schmalfuss 2003) and was mentioned by Preudhomme de Borre (1886b) as found in Belgium. However, after re-identification this specimen proved to be *A.opacum* (Preudhomme de Borre 1886a, Capart 1942).

*Armadillidiumtriviale* Schöbl, 1861 mentioned by Bellynck (1865) and Plateau (1870) appeared to be *A.vulgare* (Preudhomme de Borre 1886b, Capart 1942). This species proved to be a synonym of *A.vulgare* (Schmalfuss 2003).

*Ligidiumgermanicum* Verhoeff, 1901 was mentioned by Gysels et al. (1976), but was not mentioned on the checklists of Tavernier and Wouters (1989, 1991). However, the species appears on the checklist of Wouters et al. (2000) and is cited by Schmalfuss (2003). Wouters et al. (2000) already mentions the species as doubtful since no material has been preserved. *Ligidiumgermanicum* was deleted from this new checklist because its presence could not be confirmed.

*Eoniscussimplicissimus* Arcangeli was a specimen collected by Leruth (1937) and described as a new species to science in a new genus and family by Arcangeli (1935). Verhoeff (1937) re-examined the individual and concluded that it was a larva of a species from the millipede genus *Polydesmus* (Polk and Van Oye 1956, Polk 1957).

### Species from greenhouses

Literature on Belgian woodlice in greenhouses is very limited. Only five papers deal with inventories carried out in Belgian greenhouses and they are all from the northern part of the country. Up to date only four exotic species could be confirmed in Belgian greenhouses. They cannot be considered as part of the Belgian woodlice fauna, because of the lack of wild populations, and are not included in this checklist as Belgian species. However, they were included in previous checklists (see e.g. Capart 1942, Polk 1959a, Wouters and Tavernier 1989, 1991, Wouters et al. 2000).

The first exotic species recorded from Belgian greenhouses is *Cordioniscusstebbingi* (Patience, 1907) by Bagnall in 1908 from a greenhouse in Brussels. Polk and Van Oye (1956) mention *Trichorhinatomentosa* (Budde-Lund, 1893) from Ghent. De Smedt et al. (2017a) mention *Naguruscristatus* (Dollfus, 1889) and *Reductoniscuscostulatus* Kesselyák, 1930 both from greenhouses in Ghent (East-Flanders) and the first species also from Meise (Flemish-Brabant). In addition, Polk and Van Oye (1956) mention an individual of the genus *Rhyscotus* Budde-Lund, 1885 and De Smedt et al. (2017a) mention an individual of the genus *Synarmadillo* Dollfus, 1892. However, both specimens were lost and could not be verified.

**Greenhouse literature**: Bagnall (1907, 1908), Polk and Van Oye (1956), Kersmaekers (1973b), De Smedt et al. (2017a).

### Species to be expected

Twenty-five percent of the Belgian woodlice species were added on this new checklist and all were discovered the last 20 years, therefore it is still possible that even more species can be discovered in Belgium. Below, some species recorded in neighbouring countries and relatively close to the Belgian border are listed:

– *Porcelliogallicus* Dollfus, 1904. This species is found to be abundant in small deciduous forest fragments in agricultural areas in the north of France (Landifay-et-Bertaignemont) only 45 km from the Belgian border (De Smedt et al. 2018b). Similar habitats are also present in the southern and central part of Belgium.

– *Porcelliomontanus* Budde-Lund, 1885. Found in Germany (Wiesbaden) around 100 km from the Belgian border (edaphobase.org). Also reported from Grand Duchy of Luxembourg (Weber 2013) at only 18 km from the Belgian border. Hower, the latter record is not well documented. According to Grüner (1965) the species occurs in forest edges, under bark of trees and stone heaps. The species could therefore be expected in the south of Belgium.

– *Androniscusroseus* (C. Koch, 1838). A species closely resembling *A.dentiger* and recorded about 160 km from the Belgian border in Frankfurt (Germany) (edaphobase.org). There the species is reported from riparian habitat and forest fringe communities. The species could be overlooked since its close resemblance to *A.dentiger* and can be expected in the eastern part of the country.

– *Trachelipusratzeburgi* (Brandt, 1833). Another species that could be overlooked in Belgium because of its close resemblance to *T.rathkii*. The species occurs in all kinds of woodland and the closest records are from Herborn in Germany at 140 km from the Belgian border (edaphobase.org). Therefore, the species could be expected in the east of the country.

– *Chaetophilosciacellaria* (Dollfus, 1884). This species has recently been discovered in northern France at three localities of which two at 35 km from the Belgian border (Delasalle and Séchet 2014). The species was recorded in association with anthropogenic environments, like cemeteries. Therefore it is very likely that the species can also be found in similar habitat in Belgium.

Three of the last five new species on the Belgian list are large to medium-sized and therefore it is possible that the above-mentioned species are present and awaiting discovery.

## Conclusions

With 36 species Belgium now has a comparable amount of species, relative to its size, to neighbouring countries like the Netherlands (33 species see Berg et al. (2008) and Berg and Krediet (2017)), Great Britain (41 species see Gregory (2009) and Segers et al. (2017)) and Germany (about 50 species see edaphobase.org). France (218 species including greenhouse species see Séchet and Noël (2015)) has far more species but this is due to the additional southern species and many endemics. Despite the large amount of published papers, Belgium lagged behind in number of species recorded, probably because of the lack of an interest group, as exists for Great Britain and the Netherlands. Belgium has caught up with its neighbouring countries, although there are still some species that may be present in Belgium. Future field surveys should fill the last “blank spots” in the distribution maps and will form the base of a first distribution atlas of woodlice in Belgium. This will be a valuable way forward to understand the ecology and habitat-preference of many Western European woodlouse species.
